# Population Genetic Analysis of *Aedes aegypti* Mosquitoes From Sudan Revealed Recent Independent Colonization Events by the Two Subspecies

**DOI:** 10.3389/fgene.2022.825652

**Published:** 2022-02-14

**Authors:** Mohammed-Ahmed B. Elnour, Andrea Gloria-Soria, Rasha S. Azrag, Abeer M. Alkhaibari, Jeffrey R. Powell, Bashir Salim

**Affiliations:** ^1^ Department of Parasitology and Medical Entomology, Tropical Medicine Research Institute, National Center for Research, Khartoum, Sudan; ^2^ Department of Environmental Sciences, Center for Vector Biology and Zoonotic Diseases, The Connecticut Agricultural Experiment Station, New Haven, CT, United States; ^3^ Department of Ecology and Evolutionary Biology, Yale University, New Haven, CT, United States; ^4^ Department of Zoology, Faculty of Science, University of Khartoum, Khartoum, Sudan; ^5^ Department of Biology, Faculty of Science, University of Tabuk, Tabuk, Saudi Arabia; ^6^ Department of Parasitology, Faculty of Veterinary Medicine, University of Khartoum, Khartoum North, Sudan

**Keywords:** *Aedes aegypti*, microsatellites, genetic diversity, population genetics, Sudan

## Abstract

Increases in arbovirus outbreaks in Sudan are vectored by *Aedes aegypti,* raising the medical importance of this mosquito. We genotyped 12 microsatellite loci in four populations of *Ae. aegypti* from Sudan, two from the East and two from the West, and analyzed them together with a previously published database of 31 worldwide populations to infer population structure and investigate the demographic history of this species in Sudan. Our results revealed the presence of two genetically distinct subspecies of *Ae. aegypti* in Sudan. These are *Ae. aegypti aegypti* in Eastern Sudan and *Ae. aegypti formosus* in Western Sudan. Clustering analysis showed that mosquitoes from East Sudan are genetically homogeneous, while we found population substructure in West Sudan. In the global context our results indicate that Eastern Sudan populations are genetically closer to Asian and American populations, while Western Sudan populations are related to East and West African populations. Approximate Bayesian Computation Analysis supports a scenario in which *Ae. aegypti* entered Sudan in at least two independent occasions nearly 70–80 years ago. This study provides a baseline database that can be used to determine the likely origin of new introductions for this invasive species into Sudan. The presence of the two subspecies in the country should be consider when designing interventions, since they display different behaviors regarding epidemiologically relevant parameters, such as blood feeding preferences and ability to transmit disease.

## Introduction

*Aedes aegypti* is an invasive mosquito found across the tropical and subtropical world; and the principal vector of worldwide epidemics of arboviruses such as dengue, yellow fever, Zika, and chikungunya virus (Mourya et al., 2001; Dubrulle et al., 2009; Marcondes and Ximenes, 2016). Ancestrally, it has been suggested that this species originated in the Southwestern Indian Ocean prior to colonizing continental Africa 50–80,000 years ago (Soghigian et al., 2020) where populations still exist in tropical rainforests, with larvae breeding in tree holes and female adults taking bloodmeals from nonhuman mammals (Lounibos, 1981; McBride et al., 2014). As a result of human expansion in Africa, populations of *Ae. aegypti* evolved to become associated with human habitats, they became “domesticated” as they shifted to a more anthropophilic behavior, where larvae breed in human-generated containers and females prefer humans for bloodmeals. About 500 years ago, this human-associated form of *Ae. aegypti* left Africa, probably through the slave trade, and first invaded the New World, then subsequently Asia and the Pacific Islands, including Australia (Powell et al., 2018).

Accumulated genetic data provide evidence of strong genetic differentiation between the ancestral populations of *Ae. aegypti* in Africa and the derived populations outside of Africa (Failloux et al., 2002; Brown et al., 2011; Gloria-Soria et al., 2016; Crawford et al., 2017; Kotsakiozi et al., 2018). These two genetic groups roughly match the conventional description of the two subspecies, *Ae. aegypti formosus* (Aaf) in Africa with darker body color and *Ae. aegypti aegypti* (Aaa) outside Africa with lighter body color (Mattingly, 1957,^1967^). The *Ae. aegypti aegypti* subspecies (Aaa) that has spread throughout the tropical and subtropical world by humans (Powell and Tabachnick, 2013) is highly anthropophilic (prefers human blood-meals) (McBride et al., 2014) and is adapted to breed in human habitats “domestic” or in urban environments, is characterized by at least one pale scale on the first abdominal tergite. The ancestral form of the species in sub-Saharan Africa, *Ae. aegypti formosus* (Aaf), occurs in natural breeding habitats such as forests (Lounibos, 1981) and prefers non-human mammals for blood meals (McBride et al., 2014). These subspecies were originally described based largely on their geographic distribution, color, scaling patterns, and behavior. However, populations are highly variable for scaling pattern (McClelland, 1974; Jupp et al., 1991), so morphology does not always reflect the major ecological distinction between the two subspecies (Powell and Tabachnick, 2013).

Arboviral infections have become a major public health concern in Sudan, and they are rapidly spreading (Ahmed et al., 2020a; Elduma et al., 2020). Periodic outbreaks of arboviruses transmitted by *Ae. aegypti* have been reported, such as dengue fever in the Red Sea State (Malik et al., 2011) and in Kassala city (Abdallah et al., 2012); chikungunya in Kassala (Eldigail et al., 2020); and yellow fever in Darfur (Seidahmed et al., 2012b; Markoff, 2013; Soghaier et al., 2013; Ibrahim et al., 2015). A large-scale outbreak of chikungunya was reported in East Sudan between May 2018 and March 2019 (EMRO-WHO, 2018). Outbreaks of dengue fever occur frequently in the coastal and subcoastal areas of the country. Entomological surveillance showed that *Ae. aegypti*, was the predominant mosquito species in the area. Later, in 2015, an outbreak of dengue fever occurred among refugees in Darfur area, West Sudan, in which DENV-2 and 3 were co-circulating in the area (Ahmed et al., 2019). This was followed by a DENV-2 outbreak in 2016/17 in east Sudan (Hamid et al., 2019). This rapid change in disease burden is alarming for health authorities. Climate change, international travel and trade, and increasing human movement often arising from armed conflicts, are driving emergence of several arboviral diseases in Sudan, usually in the form of undifferentiated febrile illness (Ahmed et al., 2020b), amplified by human demographic explosion and unplanned urbanization (Ahmed et al., 2020b).

Genetic markers can be used to determine the source of invasive species and their pathways, as well as geneflow patterns, genetic composition, and demographic history of a particular population (Guillemaud et al., 2010; Maynard et al., 2017). Microsatellites, also known as simple sequence repeats (SSRs) or short tandem repeats (STRs); are short, tandemly repeated DNA motifs of 1–6 nucleotides distributed throughout eukaryotic genomes (Gadgil et al., 2017). Microsatellite markers are multi-allelic, neutral (or nearly neutral), expressed co-dominantly, distributed throughout the genome, and have been successfully used to study *Ae. aegypti* population structure (Huber et al., 2001; Brown et al., 2011; Urdaneta-Marquez and Failloux, 2011; Gloria-Soria et al., 2016; Carvajal et al., 2020). The utility of microsatellites is frequently underestimated due to the ability of researchers to genotype thousands or millions of markers across an individual genome through Next Generation Sequencing (NGS). However, the low cost of microsatellite markers makes them accessible to most research laboratories around the world. Due to their high informational content (many alleles per locus), in population genetic studies microsatellites often provide results comparable to those obtained from NGS (Gloria-Soria et al., 2016; Kotsakiozi et al., 2017; Pless et al., 2017; Carvajal et al., 2020; Lv et al., 2020), without the need of extensive computational resources for processing and analysis. An added advantage of the use of microsatellite markers is that their affordability allows for the analysis of large numbers of individuals and thus a better representation of the study populations within a budget. Moreover, data storage is inexpensive comparing to the large volumes of data generated from NGS.

At the global scale, *Ae. aegypti* genetic diversity and population genetics are well studied. (Tabachnick and Powell, 1979; Brown et al., 2011; Brown et al., 2014; Bennett et al., 2016; Gloria-Soria et al., 2016). In Sudan, however, little is known about the distribution and population genetics of the species (Elnour et al., 2020; Abuelmaali et al., 2021). The current research aims to better understand the genetic composition, population structure, and ancestral origin of *Ae. aegypti* from Sudan, which has witnessed a number of recent arboviral outbreaks, and to place Sudan *Ae. aegypti* within the global context. We compare *Ae. aegypti* populations from eastern and western Sudan and analyze their relationship with well established worldwide populations to reconstruct the most probable biogeographical scenario(s) for the invasion of *Ae. aegypti* to the Sudan.

## Materials and Methods

### Study Sites and Mosquito Collection

**FIGURE 1:**
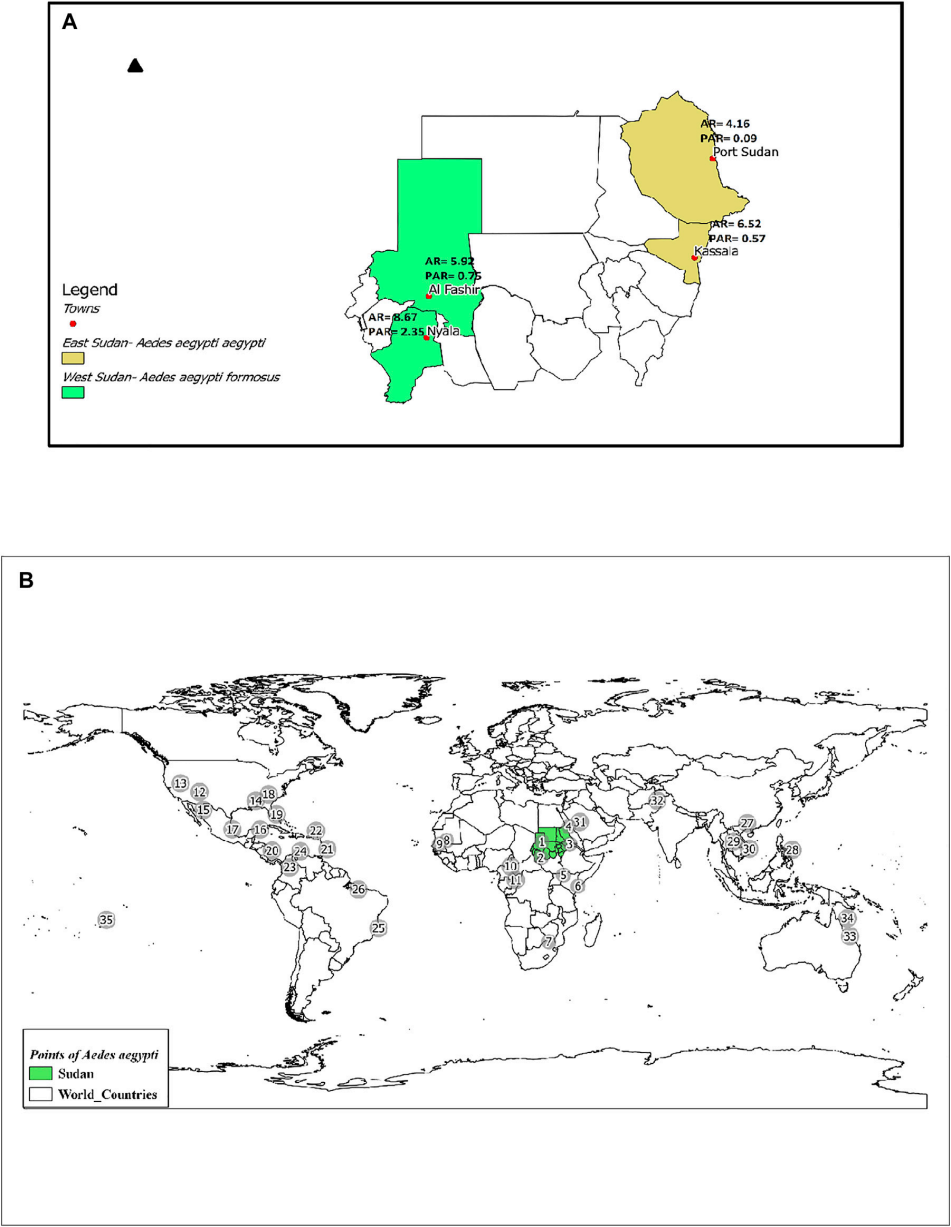
**(A)** Map of Sudan showing the locations of the two subspecies/forms of *Ae. aegypti* collected from four sites. AR = Allelic Richness and PAR = Private Allelic Richness. **(B)**. Map showing locations of *Ae. aegypti* included in this study. Population codes are as labeled in Supplementary Table S1. Shapefile downloaded from https://mapcruzin.com/

Study sites were divided into two major regions, including four cities, two cities in Eastern Sudan (Port Sudan and Kassala), and two in Western Sudan (Al Fashir and Nyala). Coordinates and geographic locations are shown in Figure 1 and Supplementary Table S1. In order to standardize the land size covered by each study area, we combined two or more small neighboring areas within each city. The maps illustrating the geographic location of collection sites were prepared using the ArcGIS software version 10.2.2 (ESRI, NC, United States) (Desktop, 2014) (Supplementary Table S1 and Figure 1).

The study sites were chosen based on their ecological features such as temperature, relative humidity, rainfall, and water storage containers in urban settings, described as the most important factors affecting *Aedes* biology (Barrera et al., 2006), in addition to the lack of basic services for economically marginalized and growing populations (Caprara et al., 2009). All sites had previous reports of arboviral diseases. Three of these study sites (Kassala, Al Fashir, and Nyala) have a tropical continental climate, characterized by a long dry season (9 months) between October and June and a short rainy season between July—September. The fourth site (Port Sudan, located in the coastal area of the Red Sea has a hot desert climate with high level of relative humidity and a short rainy season during the winter months (November—February) (Seidahmed et al., 2012a).

*Ae. aegypti* mosquitoes were collected as immature stages (larvae and pupae) using standardized sampling methods for *Ae. aegypti* surveillance (Ritchie, 2014; WHO, 2016) from various potential breeding sites mostly households from each study area in cross-sectional entomological surveys during November 2018-February 2020. The number of households per study area ranged from 10–20 with an average of 15 households within each study area and overall of 160 households. The maximum distance between households within study areas ranged from 2 to 6.5 Km. We calculated the geographical midpoint across each study area because households were widely dispersed to assign a single geographic coordinate for each study area in the subsequent genetic analysis (Figure 1 and Supplementary Table S1).

Mosquitoes included in this study came directly from the field. To avoid sampling siblings, mosquitoes were collected from multiple breeding containers across numerus sampling sites (e.g., four or more breeding sites) from any locality or residential area within each city. To avoid loss in sampling due to mortality we collected six individuals per trap per the same breeding site and reared to adults in the laboratory. Only one individual mosquito was genotyped per trap or from the same breeding site. Given that *Ae. aegypti* are “skip ovipositors” normally laying one or few eggs in multiple containers (Colton et al., 2003). The use of multiple breeding sites/traps should be sufficient to minimize sampling of siblings or individual mosquitoes from the same genetic pool. Mosquitoes were selected for genotyping to be representative of the field populations. Larvae and pupae were reared in the laboratory in favorable conditions, to avoid factors affecting adult emergence rate such as temperature, diet, predators etc., The emergence rate was very high and we selected individuals for genotyping from those that successfully emerged in the laboratory (F_0_). After their emergence, adult mosquitoes (F_0_) were identified morphologically according to the descriptions of Mattingly and Huang (Mattingly, 1957; Huang, 2004). Subsequently, they were cold -anesthetized and their wings were spread using needles to check the presence of pale scales on the first abdominal tergite under a binocular microscope. Adults mosquitoes were preserved in absolute ethanol at −20°C until DNA extraction.

### DNA Extraction and Microsatellite Genotyping

Total nucleic acids were extracted from 201 individual *Ae. aegypti* mosquitoes using the DNeasy Blood and Tissue kit (Qiagen) according to manufacturer instructions, with an additional RNAse A (Qiagen) step. Individual mosquitoes were genotyped at Jeffrey Powell’s laboratory at Yale University as described by Gloria-Soria et al. (Gloria-Soria et al., 2016). Microsatellite loci analyzed included: A1, B2, B3, A9 (tri-nucleotide repeats), and AC2, CT2, AG2, AC4, AC1, AG5, AG1, and AC5 (di-nucleotide repeats), (electronic supplementary material, Supplementary Table S2) (Slotman et al., 2007; Brown et al., 2011). Polymerase chain reactions were carried out in 10 μl reactions using the Type-it Microsatellite PCR Master Mix (Qiagen), 25 nM of each forward primer, 250 nM of each reverse primer, and 500 nM of a fluorescently labeled M13 primer to allow multiplexing (Oetting et al., 1995; Brown et al., 2011). Thermocycler conditions were: 94°C × 10′, 35 × (94°C × 30″, 54°C × 30″, 72°C × 30″), and 72°C × 5′. Microsatellite primer sequences, multiplex pairings and fluorescent primers were performed as described in Brown et al. (Brown et al., 2011). PCR products were run for fragment analysis on an Applied Biosystems 3730xl DNA Genetic Analyser with a GS 500 Rox internal size standard (Applied Biosystems) at the DNA Analysis Facility at Science Hill at Yale University. Microsatellite alleles were scored using the Geneious R11.1.4 microsatellite plugin (http://www.geneious.com). Raw microsatellite calls used for this project are provided in Supplementary Table S10 and in Vectorbase; https://vectorbase.org). Sudan genotypes can be retrieved as Vectorbase bioproject VBP0000762.

### Genetic Analysis

As larval sampling methods can lead to the collection of closely related individuals, we have used the maximum-likelihood method in ML-RELATE (Kalinowski et al., 2006) to calculate the percentages of related individuals within each population, in order to confirm that the assumption of independent genotypes was not violated. For each pair of individuals, maximum log-likelihood estimate (MLE) or R was calculated for four relatedness categories: unrelated, parent–offspring, full-siblings, and half-siblings. The MLE has a lower mean square error and performs well with a relatively small sample size (Milligan, 2003).

The exact Hardy-Weinberg equilibrium (HWE) test and linkage disequilibrium (LD) among all pairs of loci were estimated using GENEPOP v4.2.1 (Raymond and Rousset, 1995; Rousset, 2008). Significance levels for multiple testing were corrected using the Bonferroni correction. Ewens–Watterson test for neutrality (Ewens, 1972; Watterson, 1978) was calculated for all 12 loci using POPGENE 1.31 version (Yeh et al., 1999). The Ewens–Watterson test was performed for each locus separately across all populations. Briefly, the observed sum of the squared allele frequencies (observed *F*; homozygosity), was compared with the 95% confidence intervals for the expected sum of the squared allele frequencies (expected *F*). The sums of squared alleles were adjusted for sample sizes and the number of alleles. The 95% confidence intervals and standard errors for observed *F* were calculated using 1,000 simulated samples.

Measures of genetic diversity, including mean number of different alleles (*MNa*), mean number of effective alleles (*MNe*), allelic richness (*AR*), private allelic richness (*PAR*), observed heterozygosity (*Ho*), expected heterozygosity (*He*), and inbreeding coefficients (*F*_***IS***_) were estimated using Genetic Analysis in Excel (GenAlEx) version 6.3 (Peakall and Smouse, 2012). To evaluate the magnitude of genetic differentiation among sites, uncorrected and corrected pairwise F_ST_ values were calculated using Arlequin v3.5.1.3 (Excoffier and Lischer, 2010), and FreeNA (Chapuis and Estoup, 2007) respectively, with 10,000 permutations. The analysis of molecular variance (AMOVA) was performed using GenAlEx version 6.3 (Peakall and Smouse, 2012). FreeNA (Chapuis and Estoup, 2007) was also used to calculate the Cavalli Sforza and Edwards distance. Bottleneck (v1.2.2) was used to identify genetic drift, by assessing whether the loci show heterozygosity deficiency or excess (Piry et al., 1999). This analysis compares two heterozygosity scenarios: 1) the expected heterozygosity based on allele frequencies (*H*_e_) and 2) the expected heterozygosity based on observed alleles (*H*_eq_). *H*_e_ > *H*_eq_ therefore indicates a recent Bottleneck event and *H*_e_ < *H*_eq_ a recent population expansion (Cornuet and Luikart, 1996).

### Genetic Structure Analysis

In addition to the four Sudan populations genotyped in this study, we used previously reported data from 31 populations (50 individuals each, with the exception of Johannesburg (N = 18); Supplementary Table S1) from five continents, across much of *Ae. aegypti* geographic range (Brown et al., 2011; Brown et al., 2014; Gloria-Soria et al., 2016; Kotsakiozi et al., 2017; Saarman et al., 2017). These populations represent a worldwide reference panel used to establish the genetic affinities of Sudan populations with *Ae. aegypti* populations throughout the world and to identify their geographic origin(s). Bayesian clustering analysis implemented in Structure software version 2.3.2 (Pritchard et al., 2000), which identifies genetic clusters and assign individuals to these clusters with no prior information of sample location, was used to infer the most likely number of genetic clusters (K). STRUCTURE uses estimated allele frequencies to compute the likelihood that a given genotype originated from a genetic cluster and assigns each individual a probabilistic coefficient of population membership. Ancestral genetic admixture within an individual is observed when an individual has more than one population group assigned. Ten independent runs were performed for each value of K (1–10) with a burn-in phase of 200,000 iterations followed by 600,000 replications. To determine the most likely number of clusters, the commonly used ΔK statistic as developed by Evanno (Evanno et al., 2005) was calculated using the online software Structure Harvester version 0.6.93 (Earl and Vonholdt, 2012). To avoid the effects of uneven sampling in the Bayesian analysis using the software Structure version 2.3.2 (Pritchard et al., 2000), we standardized the number of individuals per study area to 50 individuals in each population. These datasets were subjected to Bayesian analysis using the same parameters and analyzed using the (Evanno et al., 2005) method. The online software http://clumpak.tau.ac.il/index.html was used to summarize and visualize the bar plots for the best K statistic identified for each dataset. To complement the genetic structure analysis, we performed Principal Component Analysis (PCA) and Discriminant Analysis of Principal Components (DAPC), using the R packages ade4 (Dray and Dufour, 2007), LEA (Frichot and François, 2015), and ADEGENET (Jombart, 2008) in R v.3.4.4 (R Core Team, 2019).

### Bottleneck Effect

We used the program BOTTLENECK (Piry et al., 1999) to test for recent population bottlenecks using the twelve loci. Wilcoxon’s signed rank test was used to compare expected heterozygosity from the Hardy–Weinberg equilibrium with predicted heterozygosity at mutation-drift equilibrium, on the basis of the observed allele number (Piry et al., 1999), as recommended for less than 20 markers. The significance level was assessed using 10,000 simulation iterations. The second method is based on allele frequency distributions. The shift in the L-shaped curve under mutation-drift equilibrium is an indicative of a recent bottleneck (Luikart et al., 1998). The program was run under the two-phase mutation model (TPM) and the Stepwise Mutation Model (SMM) model, which perform optimally for microsatellites datasets (Chakraborty and Jin, 1992; Di Rienzo et al., 1994; Slatkin, 1995). Extreme reductions in population sizes that have occurred during the last 0.2–4.0 N_e_ generations can only be detected by the program (Luikart and Cornuet, 1998). Based on the average effective population size (N_e_) for *Ae. aegypti* populations worldwide the program cannot detect bottlenecks that occurred more than ∼1,200 generations ago (Olanratmanee et al., 2013; Rašić et al., 2015; Saarman et al., 2017).

### Isolation by Distance and Spatial Autocorrelation

Pairwise geographic distances (km) among study areas of the mosquito populations were calculated using (GenAlEx) version 6.3 (Peakall and Smouse, 2012) and isolation by distance (IBD) was tested using Mantel’s test of correlation on the pairwise genetic distance *F*_*ST*_ and geographical distance (km), with 10,000 permutations, in GenAlEx version 6.3 (Peakall and Smouse, 2012).

### Demographic Analysis and Population History

We performed demographic inference of *Ae. aegypti* in Sudan following a hierarchical approach using Approximate Bayesian Computation methods (ABC), (Beaumont et al., 2002), as implemented by DIYABC v.2.0.4 (Cornuet et al., 2014). The program generates simulated datasets based on each scenario and compares them to the observed data. The scenario in which the simulated data set is closest to the observed data can then be selected based on its assigned posterior probability (P), with the most likely scenario having the higher *p* value (Cornuet et al., 2014). The scenarios were tested in six independent runs, with each run containing 30 randomly drawn individuals belonging to populations of *Ae. aegypti* from West (WS) and East Sudan (ES), Africa, and Out-of-Africa populations (first round); or from WS and ES, West Africa (WA), East Africa (EA), America, and Asia (second round). Datasets for three runs were generated by selecting individuals from different populations within each region; datasets for the other three runs were generated by drawing individuals from one representative population per region, switching populations for each of the three runs (Supplementary Table S3). Note that although for Johannesburg the maximum number of individuals that could be drawn was 18, we found no evidence that a small sample size had influence the outcome. In the first round, we tested four scenarios to determine at a broad scale the ancestral origin of East and West Sudan *Ae. aegypti* (Africa vs out-of-Africa). Scenario 1: WS and ES *Ae. aegypti* derived from Africa *Ae. aegypti*; Scenario 2: WS and ES *Ae. aegypti* derived from Out-of-Africa *Ae. aegypti*; Scenario 3: WS *Ae. aegypti* derived from Africa *Ae. aegypti* and ES *Ae. aegypti* derived from Out-of-Africa *Ae. aegypti*; Scenario 4: ES *Ae. aegypti* derived from Africa *Ae. aegypti* and WS *Ae. aegypti* derived from Out-of-Africa *Ae. aegypti*; (Figure 6, Supplementary Table S3A,B). On round two, we tested four additional scenarios derived from the best supported scenario in the first round of ABC analyses, but distinguishing Asia from America, and West and East Africa (Supplementary Table S3C,D). The best-fit scenario and confidence on the model of choice were evaluated. Divergence times were estimated in generations, with priors based on the historical record and previous studies (Powell and Tabachnick, 2013; Schaffner and Mathis, 2014; Gloria-Soria et al., 2016; Saarman et al., 2017). Given that the number of generations per year for *Ae. aegypti* is affected by the climatic conditions (Beserra et al., 2006; Marinho et al., 2016), the transformation of divergence time from generations to years for Sudan regions was estimated for tropical populations assuming an average of 10 generations per year (for details see (Beserra et al., 2006; Gloria-Soria et al., 2016; Marinho et al., 2016)). A mutation rate ranging from 9 × 10^−6^ - 1 × 10^−5^ based on previous publications and rates reported in the literature for other Diptera species was used (Schug et al., 1997; Pfeiler et al., 2013). Details on the effective population size and split time between regions used as priors for the ABC analysis are provided in Supplementary Table S3.

## Results

### Relatedness Analysis

The maximum likelihood estimation (MLE) showed that the percentage relatedness (first-degree relatives) for *Ae. aegypti* ranged between 1.6% in Nyala population and 5.3% in Port Sudan population, indicating that the great majority of individual mosquitoes are not siblings (Supplementary Table S4).

### Genetic Diversity in Sudan

In the four mosquito populations from Sudan, we identified 130 alleles distributed across 12 microsatellite loci, as shown in Supplementary Table S5. The number of alleles per locus ranged from 7 (A9) to 21 (AC5), with an average of 10.83 alleles per locus, supporting the highly polymorphic nature of the selected microsatellites (Supplementary Table S5). A summary of the genetic variation at 12 microsatellite loci by sampling location is provided in Supplementary Table S6. Average allelic richness (AR) ranged between 4.16—8.67, whereas values of private allelic richness (PAR) were between 0.09–2.35; Figure 1A. Average genetic diversity (*H*_*e*_) within populations for all loci was 0.599. Shannon’s Information Index (I) ranged from 0.056 (AG5) to 2.286 (AC5) with a mean value of 1.228 (Supplementary Table S6). Fixation Index (F_IS_) ranged from—0.0004 (B2) to 0.526 (AG1, B3) with an average value of 0.261 allele per locus (Supplementary Table S6).

After sequential Bonferroni corrections, 9 out of 48 (18.78%) within-population comparisons deviated significantly from Hardy–Weinberg Equilibrium (*p* < 0.05). Small deviations from HW may be caused by rare null alleles (Supplementary Table S7). Linkage disequilibrium (LD) test revealed that 4 of the 264 (1.51%) locus -by- locus tests remained significant after sequential Bonferroni correction, with no two loci consistently correlated across populations (Supplementary Table S8). The low level of LD reported here (1.51%) is consistent with the 12 loci being independent. Ewens-Waterson test for neutrality revealed that all microsatellite loci, except for AC5, were neutral (92% of the loci were neutral; Supplementary Table S9). This finding should not affect our results, Since it has previously been demonstrated that results generated from these microsatellite set of markers do not change when 2 of 12 loci are removed (specifically AC5 and AC9) (Brown et al., 2011). The reduced dataset produced the same pattern of population clustering and hierarchical relatedness as the full 12-locus dataset (Brown et al., 2011). Mean genetic diversity estimates over loci for each mosquito population are summarized in Table 1.

**TABLE 1:** Summary of the mean genetic diversity indices over loci for each of *Aedes aegypti* mosquito populations.

Region	Pop	N	MNa	MNe	PA	I	H_o_	H_e_	uH_e_	F
**Western Sudan**	Al Fashir	49.667	5.917	2.901	0.750	1.155	0.486	0.563	0.569	0.111
	Nyala	47.833	8.667	4.083	2.333	1.556	0.625	0.691	0.698	0.085
**Eastern Sudan**	Kassala	52.000	6.583	2.850	0.583	1.174	0.519	0.573	0.578	0.114
	Port Sudan	51.000	4.167	2.683	0.083	1.025	0.503	0.568	0.573	0.127

MNa , mean number of alleles, MNe: mean number of effective alleles, PA: private alleles, I: Shannon’s information index, H_o_: observed Heterozygosity, He: expected Heterozygosity, uHe: Unbiased Expected Heterozygosity, Fis.

Fixation index.

### Population Structure

**FIGURE 2:**
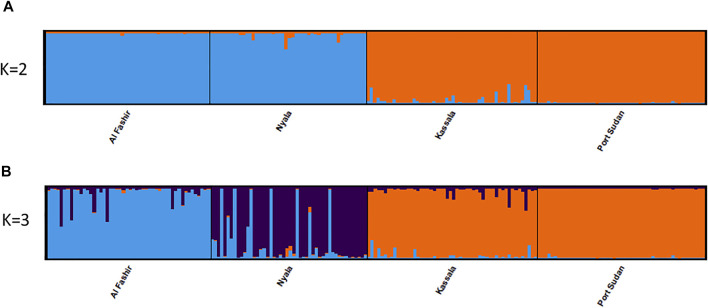
Genetic structure of *Ae. aegypti* Sudan populations. STRUCTURE bar plot indicating genetic groupings of four geographic locations in Sudan based on 12 microsatellite loci. Each vertical bar represents an individual. The height of each bar represents the probability of assignment to each of K = 2 **(A)** and of K = 3 **(B)** clusters as determined using the Delta K method. Each cluster is indicated by different colours.

**FIGURE 3:**
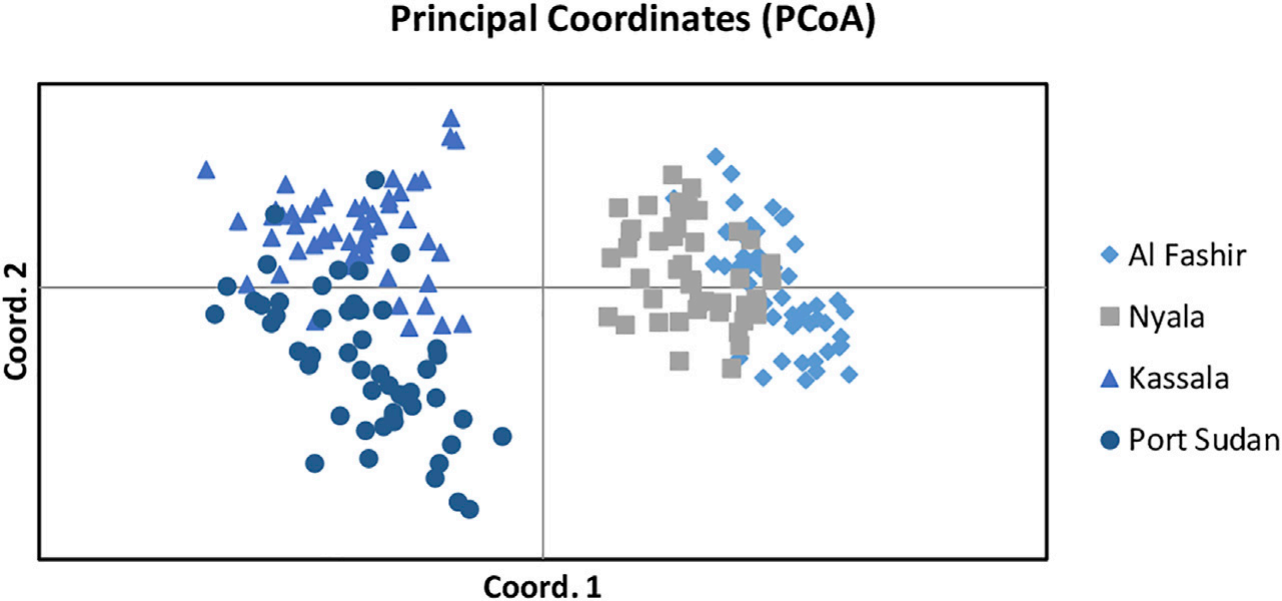
Principal Component Analysis (PCA) on the Sudan *Ae. aegypti* microsatellite dataset as implemented and plotted using the (GenAlEx) version 6.3. Populations that originated from different regions are presented with different colors.

**FIGURE 4:**
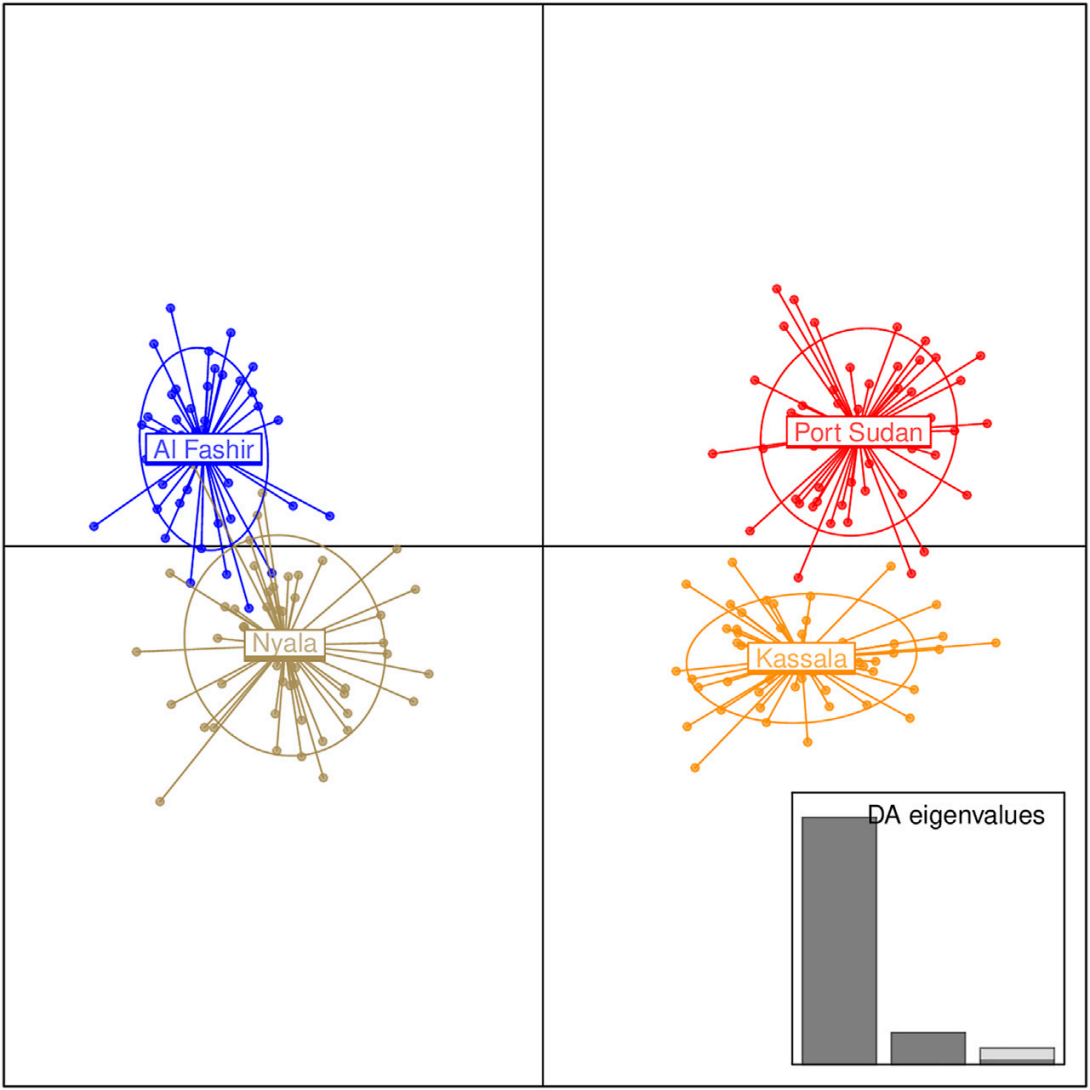
Discriminant Analysis of Principal Components (DAPC) for the *Ae. aegypti* populations from Sudan based on the microsatellite dataset. The graph represents the individuals as dots and the groups as inertia ellipses. A bar plot of eigenvalues for the discriminant analysis (DA eigenvalues) is displayed in the inset. The number of bars represent the number of discriminant functions retained in the analysis and the eigenvalues correspond to the ratio of the variance between groups over the variance within groups for each discriminant function.

Bayesian clustering analysis in STRUCTURE (Pritchard et al., 2000) and DAPC (Jombart et al., 2010) on *Ae. aegypti* from Sudan, supports the existence of two major genetic clusters (Figure 2A; Supplementary Figure S1). Population substructure was observed in Western Sudan populations (Al Fashir and Nyala), as revealed in Figure 2B. These clusters were supported by the PCA (Figure 3) and DAPC (Figure 4) analyses. AMOVA results indicate that most genetic variation in the *Ae. aegypti* populations from Sudan is found within individuals and among regions, accounting for 67 and 18% of the total variation respectively, only 5% of the total variation was attributable to differences among populations within regions populations (Table 2), suggesting a lack of genetic structure between the cities.

**TABLE 2:** Hierarchical analysis (AMOVA) of the genetic variation in the *Ae. aegypti* samples collected from different sites in Sudan.

Source of Variation	Df	Sum of Squares	Estimated Variance	Variation% (%)	*F*-statistics	*p*-value
Among regions	1	199.743	0.849	18	*F*_RT_	0.000
Among populations	2	58.524	0.251	5	*F*_SR_	0.000
Among individuals	197	804.487	0.450	10	*F*_IS_	0.000
Within individuals	201	640.000	3.184	67	*F*_IT_	0.000
Total	401	1702.754	4.733	100		

Probability estimation is based on 9,999 permutations.

For comparison among regions, Eastern and Western Sudan were considered as two different regions.

*F*_RT,_ differentiation among regions.

*F*_SR,_ differentiation among populations within group.

*F*_IS_, differentiation among individuals.

*F*_IT_, differentiation within individuals.

a Statistically significant values (*p <* 0.05).

Mosquitoes from Eastern Sudan sites were significantly differentiated from Western Sudan (Table 3), with F_ST_ values ranging from 0.050 to 0.273 among all study sites. The population pairs Al Fashir—Kassala and Al Fashir - Port Sudan had the highest F_**ST**_ values (0.273) observed, followed by Nyala - Port Sudan and Nyala - Kassala (F_ST_ >0.05 in all of the study sites). All pairwise comparisons were significant (*p* > 0.05) in all 6 pairwise comparisons. Generally, there was a lower degree of genetic differentiation among Western Sudan populations (Al Fashir-Nyala) and Eastern Sudan (Port Sudan-Kassala) than between East and West Sudan (Table 3). Genetic distance (*F*_ST_) of all four *Ae. aegypti* populations in Sudan was positively correlated with geographic distance (*R* ^2^ = 0.4272, *p* = 0.01), indicating isolation by distance.

**TABLE 3:** Pairwise F_ST_ ^a^ estimates for *Aedes aegypti* populations.

	A Fashir	Nyala	Kassala	Port Sudan
**Al Fashir**	0.000	0.050	0.269	0.262
**Nyala**	0.054	0.000	0.183	0.189
**Kassala**	0.273	0.185	0.000	0.070
**Port Sudan**	0.272	0.197	0.076	0.000

a Below the diagonal: F_ST_, values without correction for null alleles. Above the diagonal: Free NA, corrected F_ST_, values. All F_ST_, values are statistically significant (*p* < 0.05).

**FIGURE 5:**
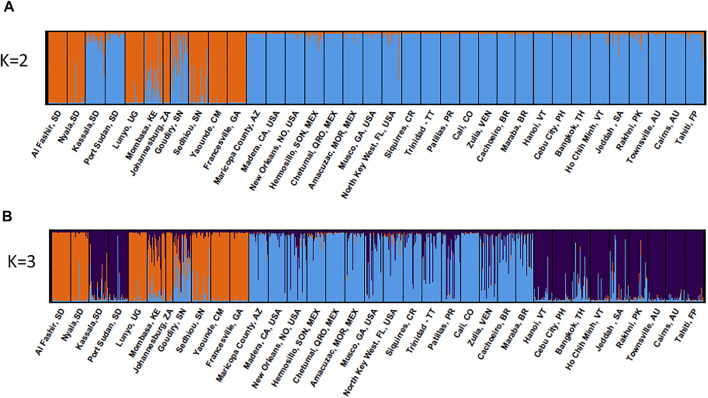
Global genetic structure of *Ae. aegypti* populations. STRUCTURE bar plot indicating genetic groupings of five continents (35 geographic locations) based on 12 microsatellite loci. Each vertical bar represents an individual. The height of each bar represents the probability of assignment to each of **(A)** K = 2 and **(B)** K = 3 clusters. The optimal number of clusters was determined using the Delta K method as K = 2. Each cluster is indicated by different colour: Aaa: orange and Aaf: blue.

Bayesian clustering analysis on the global dataset points to K = 2 being the optimum number of clusters, when evaluated using ΔK (Evanno et al., 2005) method, (Supplementary Figure S2). Western Sudan populations cluster with Aaf populations from East (Lunyo and Mombasa) and West Africa (Sédhiou, Yaounde, and Francesville), while populations from Eastern Sudan cluster with Aaa populations from Asia, North America, Australia, and the Pacific (Figure 5A). When K = 3 clusters are considered, populations from Eastern Sudan cluster with the Asian populations (Figure 5B). Different degrees of admixture are observed between the two subspecies in Sudan (Kassala), Kenya (Mombasa), and Senegal (Goudiry and Sédhiou) (Figures 5A,B).

### Inferring Population History

There was no evidence that Sudan populations of *Ae. aegypti* had undergone a recent bottleneck. The excess in heterozygosity was not significant (*p* < 0.05) in any population using the two-phase mutation and the stepwise mutation models under Wilcoxon sign-rank test and shift mode test, except for the Port Sudan population, which was significant under the one tail TPM model. However, the Mode Shift showed a normal L-shaped distribution (Table 4).

**TABLE 4:** Bottleneck analysis. Wilcoxon signed-rank tests for heterozygosity excess and mode shift under the two-phase mutation model (TPM) and stepwise mutation model (SMM). Statistically significant deviation from equilibrium is present if the value is >0.05.

Location	Population	No. samples	TPM Model	SMM Model	Mode Shift
Western Sudan	Al Fashir	50	0.740723	0.99988	Normal
	Nyala	48	0.715332	0.993286	Normal
Eastern Sudan	Kassala	52	0.866943	0.998291	Normal
	Port Sudan	51	**0.004028** ^a^	0.133057	Normal

a Significant *p-*Value < 0.05.

**FIGURE 6:**
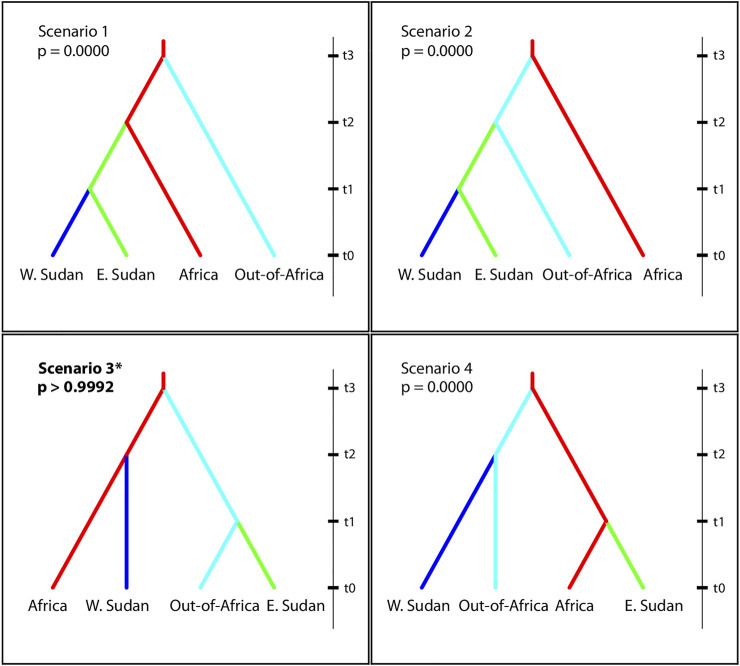
Evolutionary scenarios of *Ae. aegypti* colonization of Sudan evaluated using Approximate Bayesian Computation inference, as implemented by the DIYABC software (Cornuet et al., 2014). Scenarios include four regions: Western Sudan, Eastern Sudan, Africa, out-of-Africa, N = 30 randomly chosen individuals for each region. t0 represents the most recent time, point. Increasing values of t are not to scale and are not necessarily be in chronological order. Posterior probabilities are shown for each scenario. For more details see Materials and Methods and Supplementary Table S3.

The best supported scenario in the ABC analysis points to an independent origin of West Sudan (WS) and East Sudan (ES) populations, with 5 of six runs supporting Scenario 3: *p* > 0.9992 (Figure 6 and Supplementary Table S3A,B), in which WS has an African origin and ES originated from outside Africa. Under Scenario 3, Sudan populations would have diverged from their source at similar times, with WS diverging from surrounding African populations ∼80 years ago, and ES from populations outside Africa ∼70 years ago (considering 10 generations/year; Supplementary Table S3). A follow up ABC analysis further exploring Scenario 3 above, separating East and West Africa, America, and Asia (Supplementary Figure S3), favored a scenario in which ES *Ae. aegypti* derived from Asia and WS from West Africa *Ae. aegypti* in all 6 runs computed, although with lower posterior probabilities than those obtained from the first analysis, when the regions were combined (Scenario 1; *p* > 0.5363) (Supplementary Table S3C,D), likely due to ongoing geneflow within Africa and between Asia and America.

## Discussion

The increasing incidence of dengue, chikungunya yellow fever, and Zika virus in Sudan led us to investigate the genetic diversity and population history of *Ae. aegypti* in the country, the principal vector of arboviruses. *Ae. aegypti* has been reported broadly in several geographical areas in Sudan since the 1940s, including the eastern regions (Port Sudan and Kassala), central Sudan (Wad Medani and Khartoum), western regions (Al Fasher, and Al Junaynah), and the Nuba mountains in the south (Lewis, 1943,^1953^; Mattingly, 1957).

Our population genetics analyses on Sudan populations of *Ae. aegypti* revealed a broad range of genetic differentiation (F_ST_) among mosquito populations across study sites, which is consistent with the findings from a recent study of *Ae. aegypti* in a large Sahelian zone in Sudan that used morphology and the Cytochrome oxidase-1 mitochondrial marker (CO1) (Abuelmaali et al., 2021). Genetic differentiation between *Ae. aegypti* populations from Western Sudan and East Sudan was higher than differentiation estimated within each region and likely reflects levels of geographic isolation. Low F_ST_ within the region of western Sudan (Al Fashir- Nyala) and among eastern Sudan regions (Port Sudan—Kassala) might be explained by human-driven passive dispersal facilitating gene flow. While *Ae. aegypti* can only move hundreds of meters around their larval habitats, studies have demonstrated that *Ae. aegypti* travels long distances by taking advantage of human assisted transportation routes through land, sea, or air (Brown et al., 2014; Fonzi et al., 2015; Egizi et al., 2016). This is supported by the presence of eggs, larvae, and adults found in commercial trucks and ships (Chadee, 1992; Suleman et al., 1996); while larvae and pupae are found across transportation zones such as airports (Sukehiro et al., 2013) and ports (Suleman et al., 1996; Fonzi et al., 2015). Occasional passive limited dispersal has also been observed, up to 1 km (Reiter et al., 1995). The dichotomy of both passive and active dispersal has been a common finding among human-associated mosquitoes, especially in Southeast Asia (Olanratmanee et al., 2013; Rašić et al., 2015), and is considered as a key factor in the persistence and resurgence of mosquito-borne diseases (Paupy et al., 2004).

Our analyses revealed that West Sudan populations cluster with Aaf populations from Africa, while populations from East Sudan cluster with out-of- Africa Aaa, more specifically Asia (Figure 5B). The presence of the two subspecies of *Ae. aegypti* in Sudan is consistent with reports from parts of coastal East Africa dating back as far as the 1950s, based on morphology and habitats (Mattingly, 1957), and later supported by genetic studies (Brown et al., 2011; Gloria-Soria et al., 2016). These results are also in agreement with the findings from Abuelmaali *et al.*, (Abuelmaali et al., 2021), using the mitochondrial COI gene. The genetic differentiation observed between Sudan regions suggests there is little or no connectivity among populations of Eastern and Western Sudan, so it is not surprising that the two *Ae. aegypti* lineages remain distinct, with the exception of the admixed Kassala population (Figures 5A,B). We find that Western Sudan populations of Aaf are more genetically differentiated and structured than Aaa populations in Eastern Sudan. This is in contrast to what was observed by Gloria-Soria *et al.*, (Gloria-Soria et al., 2016), at the global scale, with low genetic differentiation observed in Aaf from Africa, relative to Aaa populations outside Africa. The difference could be explained by *Ae. aegypti aegypti* being introduced to East Sudan from a genetically homogeneous source from outside Africa and the persistence of gene flow within the region.

The demographic analysis support that the two major genetic groups of *Ae. aegypti* in Sudan evolved from two independent introductions (Figure 6, Supplementary Figure S3, and Supplementary Table S3), one from Aaf from within Africa and one Aaa likely from Asia. The estimated time of divergence between Sudan *Ae. aegypti* and its ancestral populations (∼800 generations) is in remarkable agreement with historical records. Assuming ∼10 generations/year, our results are consistent with the Sudan populations originating between 70 and 80 years ago. These findings may be in part explained by the historical migrations from West Africa to the Western Sudan either through pilgrimage to Saudi Arabia or through the trade with West and East Africa. Asian countries might be an important source of mosquito vectors and possibly dengue virus serotypes into Sudan, driven by the international trade and travel to these countries through the major port of Sudan in the Red Sea (Port Sudan).

In this study, we collected Aaf from urban and pre-urban areas in Western Sudan (Al Fashir and Nyala) cities. *Ae. aegypti* populations in Africa are known to historically bred almost entirely in forests. Today, populations of *Ae. aegypti* in Africa can be found in urban habitats (Paupy et al., 2008; Kamgang et al., 2013; Abuelmaali et al., 2021), even if they fall morphologically and genetically into Aaf. Aaf has been previously identified in African urban areas in Senegal (Huber et al., 2008; Paupy et al., 2008; Sylla et al., 2009; Gloria-Soria et al., 2016). Our findings are consistent with the conclusion from Brown *et al* (Brown et al., 2011) suggesting multiple independent domestication events taking place in Africa, most probably as a response to expanding urbanization (Gloria-Soria et al., 2016). The presence of Aaf and its association with human dwellings in Western Sudan suggests that Aaf might be the sole vector of arboviral diseases in this region (Lewis, 1953; Abuelmaali et al., 2021).

Our results recapitulate the regional genetic structure detected by Abuelmaali *et al.*, (Abuelmaali et al., 2021), and expand these findings by using 12 highly polymorphic markers and a global dataset to elucidate the demographic history of Sudan populations. Future studies should focus on increasing the number of sites from across Sudan and its African region to generate a fine-scale picture of *Ae. aegypti* genetic structure and demographic history of the country. Unfortunately, more extensive sampling is currently limited by the political conflicts in the region. Here, we have shown that microsatellites have sufficient resolution to uncover the large-scale population structure in an area where the research budget is limited. However, they did not provide enough resolution to narrow down the precise source of the introductions that led to Sudan’s populations. Many more genome-wide markers will be needed in the near future to address this question, together with a larger reference panel that includes nearby regions. At the moment, the costs associated with this kind of study are high and beyond our budget.

Our findings provide base-line data of the current population genetic status of the mosquito vector, *Ae. aegypti* in Sudan, genetic diversity and connectivity patterns among regions that can be used to monitor new introductions, changes in gene flow patterns, and the success of control strategies. Populations of *Ae. aegypti* considerably vary in their competence to transmit arboviruses (Tabachnick et al., 1985; Black et al., 2002; Sim et al., 2013) and resistance to insecticides (Montella et al., 2007; Linss et al., 2014). The presence of the two subspecies in Sudan should be considered when designing any vector control intervention, since they differ in their behavior and ability to transmit the disease (Gouck, 1972; Lounibos, 1981; Powell and Tabachnick, 2013; McBride et al., 2014). Genetic modification that relies on standing genetic variation in populations (Powell and Tabachnick, 2014) will need to be specific to the target population and thus if the country pursues innovative vector control approaches, our findings will be crucial to the success of the control program. Finally, human mediated transportation and migrations are facilitating long distance vector dispersal could result in admixture of forest-adapted and urban-adapted populations leading to increased adaptive flexibility, with implications for disease transmission and control.

## Data Availability

The datasets presented in this study can be found in online repositories. The names of the repository/repositories and accession number(s) can be found in the article/Supplementary Material.

## Supplementary Material

Supplementary Figure S1 Scatter plots of ΔK **(A,C)** and Log probability of the data **(B,D)** for the two groups of *Ae. aegypti* populations. ΔK plots are based on the rate of change in the log probability of the data between successive K values. According to Evanno et al. (2005), the most probable (optimum) number of genetically differentiated groups using all individuals across mosquito populations is K = 2 **(D)** for Sudan samples.

Supplementary Figure S2 Scatter plots of ΔK **(A,C)** and Log probability of the data **(B,D)** for the two groups of *Ae. aegypti* populations for Sudan plus Global populations. ΔK plots are based on the rate of change in the log probability of the data between successive K values. According to Evanno et al. (2005), the most probable (optimum) number of genetically differentiated groups using all individuals across mosquito populations is K = 2 **(D)** for Global population.

Supplementary Figure S3 Evolutionary scenarios of *Aedes aegypti* colonization of Sudan evaluated using Approximate Bayesian Computation inference, as implemented by the DIYABC software (Cornuet et al., 2014). Round 2, Scenarios include four regions: Western Sudan, Eastern Sudan, Africa, out-of-Africa, *N* = 30 randomly chosen individuals for each region. t0 represents the most recent time, point. Increasing values of t are not to scale and are not necessarily be in chronological order. Posterior probabilities are shown for each scenario. For more details, see *Materials and Methods* and Supplementary Table S2.

Supplementary Table S1 Detailed collection information of *Aedes aegypti* populations including geographic region, number of individuals per sample, geographic coordinates of each location, and number of generations reared in the laboratory previous to genetic analysis.

Supplementary Table S2 Microsatellite primer sequences, multiplex pairings and fluorescent primers.

Supplementary Table S3
**(A)** Priors and posteriors (logistic approach) for the ABC analysis testing scenarios on the history of Sudan *Aedes aegypti*. (Random sampled individuals from Africa and Out-of-Africa [Asia + America] pools). **(B)** Priors and posteriors (logistic approach) for the ABC analysis testing scenarios on the history Sudan *Aedes aegypti*. (Representative populations from Africa and Out-of-Africa [Asia/America]). **(C)** Priors and posteriors (logistic approach) for the ABC analysis testing scenarios on the history of Sudan *Aedes aegypti* considering East and West Africa, and America and Asia, as separate groups (Random sampled individuals from W and E Africa, Asia and America pools). **(D)** Priors and posteriors (logistic approach) for the ABC analysis testing scenarios on the history of Sudan *Aedes aegypti* considering East and West Africa, and America and Asia, as separate groups (Populations).

Supplementary Table S4 Maximum Likelihood estimates (MLE)/ Relatedness for each population of *Ae. aegypti* in Sudan.

Supplementary Table S5 Allele frequencies by population and total for codominant data.

Supplementary Table S6 Summary of the genetic variation at 12 microsatellite loci by sampling location.

Supplementary Table S7 Summary of chi-square tests for Hardy-Weinberg equilibrium.

Supplementary Table S8 Genotypic linkage disequilibrium locus by-locus for all microsatellites loci for *Aedes aegypti* Sudan populations.

Supplementary Table S9 The overall Ewens-Watterson test for Neutrality.

Supplementary Table S10 Raw microsatellites calls/ genotypes used for this study.
