# Supplementary material for: Population Genetic Analysis of Aedes aegypti Mosquitoes From Sudan Revealed Recent Independent Colonization Events by the Two Subspecies
Source: Front Genet. 2022 Feb 14;13:825652. doi: 10.3389/fgene.2022.825652 (PMC8889412; doi:10.3389/fgene.2022.825652)
Supplement: Supplementary file 2 [file Table9.DOCX]

| **Table S9:** The overall Ewens-Watterson test for Neutrality   \| **Locus** \| **n** \| **K** \| **Obs. F** \| **Min F** \| **Max F** \| **Mean*** \| **SE*** \| **L95*** \| **U95*** \| \| --- \| --- \| --- \| --- \| --- \| --- \| --- \| --- \| --- \| --- \| \| AC1 \| 402 \| 13 \| 0.2210 \| 0.0769 \| 0.9421 \| 0.2816 \| 0.0110 \| 0.1506 \| 0.5620 \| \| AC2 \| 402 \| 12 \| 0.4972 \| 0.0833 \| 0.9468 \| 0.2936 \| 0.0120 \| 0.1508 \| 0.5975 \| \| AC4 \| 402 \| 8 \| 0.4968 \| 0.1250 \| 0.9658 \| 0.4167 \| 0.0229 \| 0.2153 \| 0.7872 \| \| AC5 \| 402 \| 21 \| 0.0889 \| 0.0476 \| 0.9054 \| 0.1753 \| 0.0042 \| 0.0990 \| 0.3500 \| \| CT2 \| 402 \| 8 \| 0.2589 \| 0.1250 \| 0.9658 \| 0.4183 \| 0.0243 \| 0.2206 \| 0.8058 \| \| AG1 \| 402 \| 9 \| 0.2044 \| 0.1111 \| 0.9610 \| 0.3862 \| 0.0212 \| 0.2014 \| 0.7636 \| \| AG2 \| 402 \| 16 \| 0.1989 \| 0.0625 \| 0.9282 \| 0.2291 \| 0.0070 \| 0.1245 \| 0.4471 \| \| AG5 \| 402 \| 8 \| 0.3671 \| 0.1250 \| 0.9658 \| 0.4127 \| 0.0221 \| 0.2121 \| 0.7733 \| \| A1 \| 402 \| 11 \| 0.1900 \| 0.0909 \| 0.9515 \| 0.3196 \| 0.0146 \| 0.1702 \| 0.6297 \| \| A9 \| 402 \| 7 \| 0.2724 \| 0.1429 \| 0.9706 \| 0.4621 \| 0.0296 \| 0.2271 \| 0.8624 \| \| B2 \| 402 \| 8 \| 0.2591 \| 0.1250 \| 0.9658 \| 0.4169 \| 0.0231 \| 0.2165 \| 0.7938 \| \| B3 \| 402 \| 9 \| 0.4973 \| 0.1111 \| 0.9598 \| 0.3812 \| 0.0200 \| 0.1941 \| 0.7392 \|   * These statistics were calculated using 1000 simulated samples. |  |  |  |  |  |  |  |  |  |  |  |  |  |  |  |  |  |  |  |  |
| --- | --- | --- | --- | --- | --- | --- | --- | --- | --- | --- | --- | --- | --- | --- | --- | --- | --- | --- | --- | --- | --- | --- | --- | --- | --- | --- | --- | --- | --- | --- | --- | --- | --- | --- | --- | --- | --- | --- | --- | --- | --- | --- | --- | --- | --- | --- | --- | --- | --- | --- | --- | --- | --- | --- | --- | --- | --- | --- | --- | --- | --- | --- | --- | --- | --- | --- | --- | --- | --- | --- | --- | --- | --- | --- | --- | --- | --- | --- | --- | --- | --- | --- | --- | --- | --- | --- | --- | --- | --- | --- | --- | --- | --- | --- | --- | --- | --- | --- | --- | --- | --- | --- | --- | --- | --- | --- | --- | --- | --- | --- | --- | --- | --- | --- | --- | --- | --- | --- | --- | --- | --- | --- | --- | --- | --- | --- | --- | --- | --- | --- | --- | --- | --- | --- | --- | --- | --- | --- | --- | --- | --- | --- | --- | --- | --- | --- | --- | --- | --- | --- |
