# Supplementary figures and images for: Population Genetic Analysis of Aedes aegypti Mosquitoes From Sudan Revealed Recent Independent Colonization Events by the Two Subspecies
Source: Front Genet. 2022 Feb 14;13:825652. doi: 10.3389/fgene.2022.825652 (PMC8889412; doi:10.3389/fgene.2022.825652)

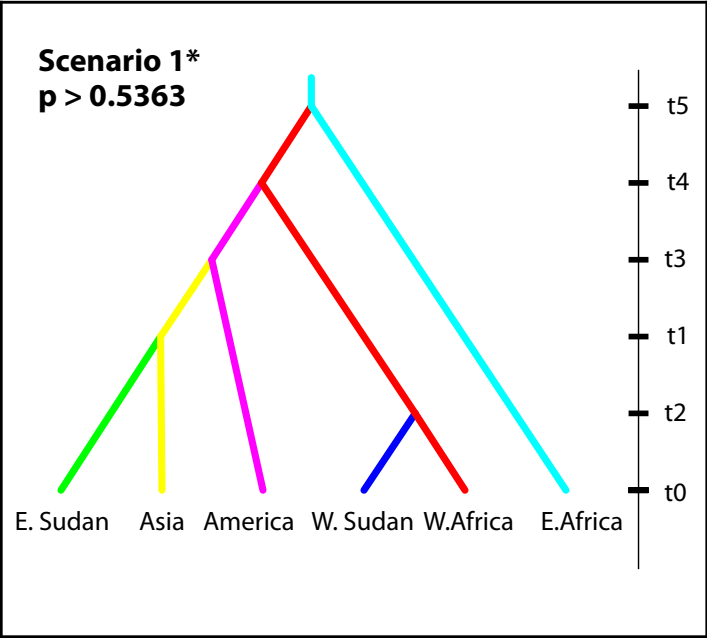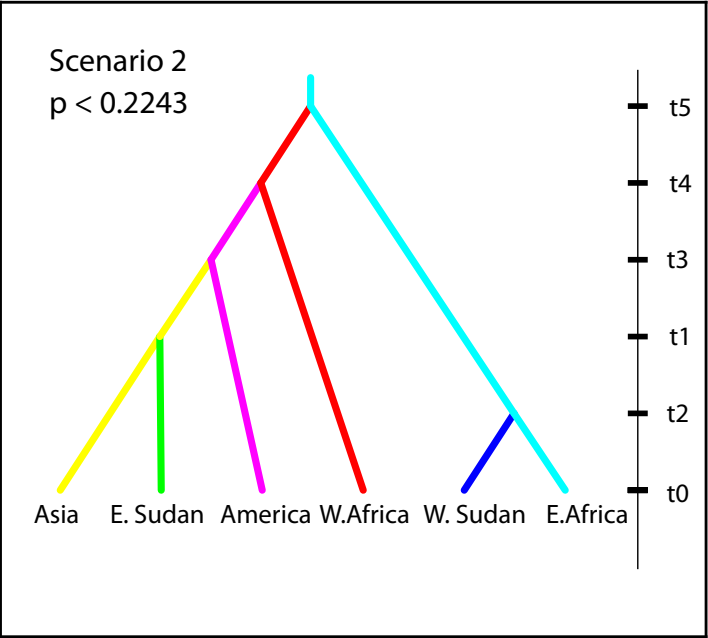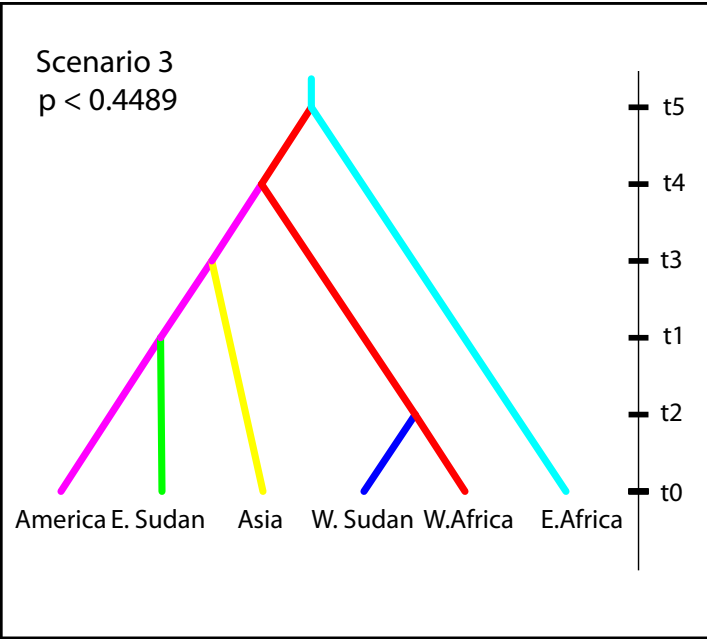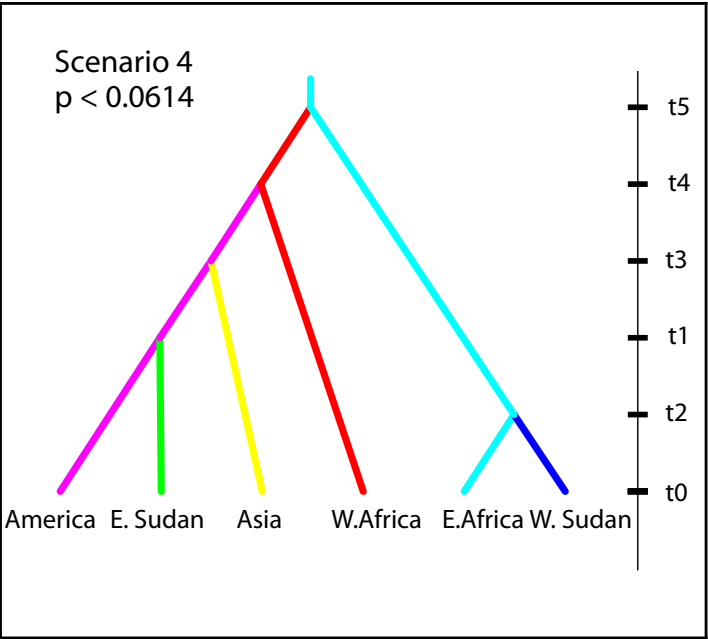

Supplement: Supplementary file 3 [file DataSheet1.PDF]

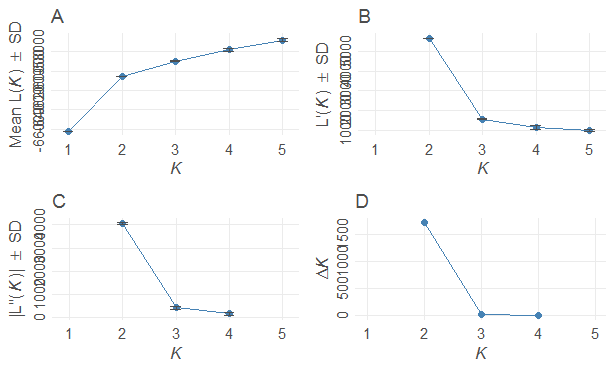

Supplement: Supplementary file 6 [file Image2.PNG]

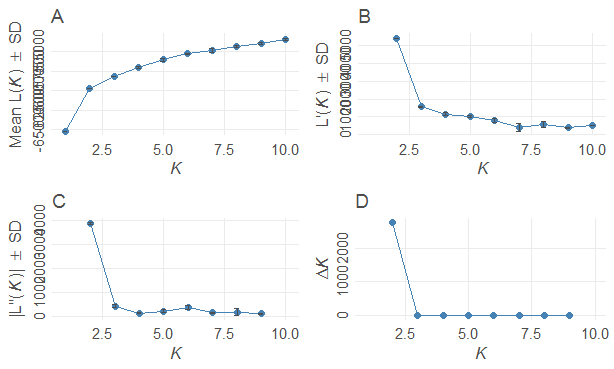

Supplement: Supplementary file 8 [file Image1.PNG]
