# Supplementary material for: Population Genetic Analysis of Aedes aegypti Mosquitoes From Sudan Revealed Recent Independent Colonization Events by the Two Subspecies
Source: Front Genet. 2022 Feb 14;13:825652. doi: 10.3389/fgene.2022.825652 (PMC8889412; doi:10.3389/fgene.2022.825652)
Supplement: Supplementary file 5 [file Table3.DOCX]

#### Table S3A. Priors and posteriors (logistic approach) for the ABC analysis testing scenarios on the history of Sudan *Aedes aegypti.* (Random sampled individuals from Africa and Out-of-Africa [Asia + America] pools).

| **Parameter** | **Details** | **Prior** | **Posterior** |
| --- | --- | --- | --- |
|  |  |  |  |
| Colonization scenarios (3 independent replicas) | Scenario 1 –  W and E Sudan *Ae. aegypti* derived from Africa *Ae. aegypti*  Scenario 2–  W and E Sudan *Ae. aegypti* derived from Out of Africa *Ae. aegypti*  **Scenario 3 –**  **W Sudan *Ae. aegypti* derived from Africa *Ae. aegypti* and E Sudan *Ae. aegypti* derived from Out of Africa *Ae. aegypti***  Scenario 4 –  E Sudan *Ae. aegypti* derived from Africa *Ae. aegypti* and W Sudan *Ae. aegypti* derived from Out of Africa *Ae. aegypti* | N/A  N/A  N/A  N/A | A) 0.0000 [0.0000 – 0.0025]  B) 0.0000 [0.0000 – 0.0001]  C) 0.0000 [0.0000 – 0.0001]  A) 0.0000 [0.0000 – 0.0000]  B) 0.0000 [0.0000 – 0.0000]  C) 0.0000 [0.0000 – 0.0000]  **A) 0.9992 [0.9975** – **1.0000]**  **B)** **1.0000 [0.9999** – **1.0000]**  C) **1.0000 [0.9999** – **1.0000]**  A) 0.0000 [0.0000 – 0.0000]  B) 0.0000 [0.0000 – 0.0000]  C) 0.0000 [0.0000 – 0.0000] |
|  |  |  |  |
| **SCENARIO 3** |  |  |  |
| Effective population size | W Sudan  E Sudan  Africa  Out of Africa (America + Asia) | 10 –10,000  10 – 10,000  100 – 450,000  100 – 400,000 | A) 9,560 [8,640 – 9,980]  B) 9,370 [8,090 – 9,970]  C) 9,300 [7,860 – 9,960]  A) 7,720 [4,480 – 9,770]  B) 7,590 [4,280 – 9,740]  C) 7,810 [4,600 – 9,800]  A) 197,000 [67,400 – 391,000]  B) 73,400 [13,600 – 242,000]  C) 96,500 [20,300 – 287,000]  A) 71,900 [29,500 – 165,000]  B) 43,000 [16,200 – 99,800]  C) 38,600 [14,400 – 90,500] |
|  |  |  |  |
| ^1^Split time | E Sudan from Out of Africa (America + Asia)  W Sudan from Africa  Out of Africa (America + Asia) from Africa | 100 – 6,000  100 – 6,000  4,500 – 6000 | A) 767 [205 – 1,480]  B) 469 [155 – 896]  C) 748 [202 – 1,430]  A) 841 [408 – 1,320]  B) 672 [336 – 1,060]  C) 810 [391 – 1,290]  A) 5,140 [4,550 – 5,880]  B) 5,170 [4,560 – 5,900]  C) 5,300 [4,590 – 5,940] |
|  |  |  |  |
| Mutation rate | Microsatellite –Stepwise Mutation Model (SMM) | 9x10^-06^-1x10^-05^ | A) 9.52x10^-06^ [9.07x10^-06^ - 9.96x10^-06^]  B) 9.47x10^-06^ [9.03x10^-06^ – 9.92x10^-06^]  C) B) 9.48x10^-06^ [9.03x10^-06^ – 9.92x10^-05^] |
|  |  |  |  |
| Confidence | Type I error  (Simulated under scenario 3)  Type II error  (Simulated under scenario 1)  Type II error  (Simulated under scenario 2)  Type II error  (Simulated under scenario 4) | N/A  N/A  N/A  N/A | A) 0.119  B) 0.148  C) 0.172  A) 0.052  B) 0.038  C) 0.048  A) 0.064  B) 0.042  C) 0.064  A) 0.132  B) 0.118  C) 0.132 |

^1^ Time in generations (10 generations / year).

W: West

E: East

#### Table S3B. Priors and posteriors (logistic approach) for the ABC analysis testing scenarios on the history Sudan *Aedes aegypti.* (Representative populations from Africa and Out-of-Africa [Asia/America]).

| **Parameter** | **Details** | | **Prior** | **Posterior** |
| --- | --- | --- | --- | --- |
|  |  | |  |  |
| Colonization scenarios:   1. W Sudan [Al Fashir], E Sudan [Kassala], Mombasa [Kenya], New Orleans LA [] 2. W Sudan [Nyala], E Sudan [Port Sudan], Lunyo [Uganda], Cali [Colombia] 3. W Sudan [Al Fashir], E Sudan [Port Sudan], Johannesburg [South Africa], Jeddah [Saudi Arabia] | Scenario 1 –  W and E Sudan *Ae. aegypti* derived from Africa *Ae. aegypti*  Scenario 2–  W and E Sudan *Ae. aegypti* derived from Out of Africa *Ae. aegypti*  **Scenario 3 –**  **W Sudan *Ae. aegypti* derived from Africa *Ae. aegypti* and E Sudan *Ae. aegypti* derived from Out of Africa *Ae. aegypti***  Scenario 4 –  E Sudan *Ae. aegypti* derived from Africa *Ae. aegypti* and W Sudan *Ae. aegypti* derived from Out of Africa *Ae. aegypti* | | N/A  N/A  N/A  N/A | **A) 0.8861 [0.6947 – 1.0000]**  B) 0.0000 [0.0000 – 0.0000]  C) 0.0000 [0.0000 – 0.0000]  A) 0.0051 [0.0000 – 1.0000]  B) 0.0000 [0.0000 – 0.0000]  C) 0.0000 [0.0000 – 0.0000]  A) 0.1082 [0.0000 – 1.0000**]**  **B) 1.0000 [1.0000 – 1.0000]**  **C) 1.0000 [1.0000 – 1.0000]**  A) 0.0006 [0.0000 – 1.0000]  B) 0.0000 [0.0000 – 0.0000]  C) 0.0000 [0.0000 – 0.0000] |
|  |  | |  |  |
| **SCENARIO 3** |  |  | |  |
| Effective population size | W Sudan  E Sudan  Africa  Out of Africa (America + Asia) | 10 –10,000  10 – 10,000  100 – 450,000  100 – 400,000 | | A) 9,810[9,390 – 9,990]  B) 9,920 [9,740 – 10,000]  C) 9,080[7,280 – 9,950]  A) 9,740[9,160 – 9,990]  B) 7,980[4,900 – 9,790]  C) 5,720[2,070 – 9,350]  A) 35,700[5,230 – 132,000]  B) 15,300[1,830 – 53,200]  C) 31,500[4,280 – 116,000]  A) 44,700[17,300 – 101,000]  B) 9,200[3,560 – 18,800]  C) 26,200[11,900 – 53,100] |
|  |  |  | |  |
| ^1^Split time | E Sudan from Out of Africa (America + Asia)  W Sudan from Africa  Out of Africa (America + Asia) from Africa | 100 – 6,000  100 – 6,000  4,500 – 6000 | | A) 2,130[572 – 3,490]  B) 1,040[249 – 2,030]  C) 656[ 207 – 1,230]  A) 3,100[1,790 – 4,130]  B) 1,250[597 – 1,960]  C) 1,550[719 – 2,460]  A) 5,020[4,530 – 5,820]  B) 5,030[4,530 – 5,830]  C) 5,390[4,620 – 5,960] |
|  |  |  | |  |
| Mutation rate | Microsatellite –Stepwise Mutation Model (SMM) | 9x10^-06^-1x10^-05^ | | A) 9.63x10^-06^ [9.17x10^-06^ - 1.00x10^-05^]  B) 9.52x10^-06^ [9.07x10^-06^ - 9.96x10^-06^]  C) 9.50x10^-06^ [9.06x10^-06^ - 9.94x10^-06^] |
|  |  |  | |  |
| Confidence | Type I error  (Simulated under scenario 3)  Type II error  (Simulated under scenario 1)  Type II error  (Simulated under scenario 2)  Type II error  (Simulated under scenario 4) | N/A  N/A  N/A  N/A | | A) 0.152  B) 0.184  C) 0.210  A) 0.003  B) 0.042  C) 0.036  A) 0.084  B) 0.056  C) 0.07  A) 0.156  B) 0.116  C) 0.146 |

^1^ Time in generations (10 generations / year).

W: West

E: East

#### Table S3C. Priors and posteriors (logistic approach) for the ABC analysis testing scenarios on the history of Sudan *Aedes aegypti* considering East and West Africa, and America and Asia, as separate groups (Random sampled individuals from W and E Africa, Asia and America pools).

| **Parameter** | **Details** | **Prior** | **Posterior** |
| --- | --- | --- | --- |
| Colonization scenarios (3 independent replicas) | **Scenario 1 –**  **E Sudan *Ae. aegypti* derived from Asia & W Sudan from W Africa *Ae. aegypti***  Scenario 2–  E Sudan *Ae. aegypti* derived Asia & W Sudan from E Africa *Ae. aegypti*  Scenario 3 –  E Sudan *Ae. aegypti* derived from America & W Sudan from W Africa *Ae. aegypti*  Scenario 4 –  E Sudan *Ae. aegypti* derived from America & W Sudan from E Africa *Ae. aegypti* | N/A  N/A  N/A  N/A | **A) 0.6641 [0.3686 – 0.9596]**  **B) 0.5363 [0.2200 – 0.8526]**  **C) 0.5458 [0.3680 – 0.7235]**  A) 0.0180 [0.0000 – 0.6825]  B) 0.0769 [0.0000 – 0.8876]  C) 0.0016 [0.0000 – 0.9107]  A) 0.4093 [0.0000 – 0.8453]  B) 0.3254 [0.1095 – 0.5414]  C) 0.4489 [0.2393 – 0.6584]  A) 0.0085 [0.0000 – 1.0000]  B) 0.0614 [0.0000 – 1.0000]  C) 0.0038 [0.0000 – 1.0000] |

| **SCENARIO 1** |  |  |  |
| --- | --- | --- | --- |
| Effective population size | W Sudan  E Sudan  West Africa  East Africa  America  Asia | 10 –10,000  10 – 10,000  100 – 450,000  100 – 450,000  100 – 400,000  100 – 400,000 | A) 7,680[3,800 – 9,850]  B) 9,030 [6,840 – 9,960]  C) 6,450 [2,190 – 9,680]  A) 5,260 [1,480 – 8,990]  B) 5,970 [1,970 – 9,330]  C) 7,520 [3,800 – 9,700]  A)181,000 [69,600 – 357,000]  B)108,000 [31,800 – 276,000]  C)161,000 [57,700 – 339,000]  A) 74,300 [35,500 – 136,000]  B) 53,100 [23,600 – 100,000]  C) 61,100 [28,100 – 113,000]  A) 33,140 [5,713 – 124,000]  B) 28,600 [5,420 – 107,000]  C) 26,500 [4,590 – 99,000]  A) 171,000 [46,400 – 360,000]  B) 211,000 [68,300 – 378,000]  C) 115,000 [20,100 – 324,000] |
|  |  |  |  |
| ^1^Split time | E Sudan from Asia  W Sudan from West Africa  Asia from America  America from West Africa  West Africa from East Africa | 10 – 15,000  10 – 15,000  100 – 3,500  4,500 – 6000  10,000 – 1,000,000 | A) 797 [173 – 1,620]  B) 1,280 [281 – 2,530]  C) 2,340 [561 – 4,320]  A) 1,080 [211 – 2,230]  B) 2,010 [412 – 4,140]  C) 1,400 [261 – 3,010]  A) 1,350 [384 – 2,920]  B) 1,850 [691 – 3,190]  C) 1,840 [657 – 3,190]  A) 5,510 [4,710 – 5,970]  B) 5,500 [4,700 – 5,970]  C) 5,450 [4,660 – 5,960]  A) 14,900 [11,000 – 20,700]  B) 12,100 [10,400 – 14,700]  C) 11,700 [10,300 – 13,700] |
|  |  |  |  |
| Mutation rate | Microsatellite –Stepwise Mutation Model (SMM) | 9x10^-06^-1x10^-05^ | A) 9,52 x10^-06^ [9.08 x10^-06^ – 9.98 x10^-06^]  B) 9.53 x10^-06^ [x10^-06^ –x10^-06^]  C) 9.42 x10^-06^ [9.00 x10^-06^ – 9.88 x10^-06^] |
|  |  |  |  |
| Confidence | Type I error  (Simulated under scenario 1)  Type II error  (Simulated under scenario 2)  Type II error  (Simulated under scenario 3)  Type II error  (Simulated under scenario 4) | N/A  N/A  N/A  N/A | A) 0.322  B) 0.288  C) 0.280  A) 0  B) 0.004  C) 0.006  A) 0.262  B) 0.312  C) 0.282  A) 0.002  B) 0.004  C) 0.000 |

^1^ Time in generations (10 generations / year).

W: West

E: East

#### Table S3D Priors and posteriors (logistic approach) for the ABC analysis testing scenarios on the history of Sudan *Aedes aegypti* considering East and West Africa, and America and Asia, as separate groups (Populations).

| **Parameter** | **Details** | **Prior** | **Posterior** |
| --- | --- | --- | --- |
|  |  |  |  |
| Colonization scenarios:   1. Al Fashir [W Sudan], Port Sudan [E Sudan], Lope [Gabon], Johannesburg [South Africa], New Orleans, [USA], Jeddah [Saudi Arabia] 2. Al Fashir [W Sudan], Kassala [E Sudan], Yaounde [Cameroon], Mombasa [Kenya], Cali [Colombia], Hanoi [Vietnam] 3. Nyala [W Sudan], Kassala [E Sudan], Lunyo [Uganda], Mombasa [Kenya] Georgia [USA], Bangkok [Thailand] | **Scenario 1 –**  **E Sudan *Ae. aegypti* derived from Asia & W Sudan from W Africa *Ae. aegypti***  Scenario 2–  E Sudan *Ae. aegypti* derived Asia & W Sudan from E Africa *Ae. aegypti*  Scenario 3 –  E Sudan *Ae. aegypti* derived from America & W Sudan from W Africa *Ae. aegypti*  Scenario 4 –  E Sudan *Ae. aegypti* derived from America & W Sudan from E Africa *Ae. aegypti* | N/A  N/A  N/A  N/A | **A) 0.7330 [0.4515 – 1.0000]**  **B) 0.5495 [0.3815 – 0.7176]**  **C) 0.7809 [0.6412 – 0.9206]**  A) 0.2243 [0.0000 – 0.4990]  B) 0.0763 [0.0000 – 0.2941]  C) 0.0109 [0.0000 – 0.0496]  A) 0.0248 [0.0000 – 0.1040]  B) 0.3237 [0.0627 – 0.5847]  C) 0.1910 [0.0695 – 0.3126]  A) 0.0179 [0.0000 – 0.7222]  B) 0.0504 [0.0000 – 0.2579]  C) 0.0171 [0.0000 – 0.0692] |
|  |  |  |  |

| **SCENARIO 1** |  |  |  |
| --- | --- | --- | --- |
| Effective population size | W Sudan  E Sudan  West Africa  East Africa  America  Asia | 10 –10,000  10 – 10,000  100 – 450,000  100 – 450,000  100 – 400,000  100 – 400,000 | A) 8,310 [5,230 – 9,890]  B) 8,270 [5,220 – 9,880]  C) 9,630 [8,760 – 9,990]  A) 4,490 [1,060 – 8,700]  B) 8,190 [5,010 – 9,830]  C) 9,050 [7,010 – 9.920]  A) 99,000 [32,600 – 230,000]  B) 51,400 [12,700 – 142,000]  C) 37,400 [7,460 – 117,000]  A) 63,800 [27,000 – 123,000]  B) 24,400 [8,060 – 51,900]  C) 20,400 [7,590 – 41,100]  A) 46,100 [15,000 – 129,000]  B) 8,440 [2,350 – 19,100]  C) 30,500 [6,540 – 106,000]  A) 92,700 [12,000 – 293,000]  B) 72,600 [6,850 – 259,000]  C) 100,000 [14,900 – 304,000] |
|  |  |  |  |
| ^1^Split time | E Sudan from Asia  W Sudan from West Africa  Asia from America  America from West Africa  West Africa from East Africa | 10 – 15,000  10 – 15,000  100 – 3,500  4,500 – 6000  10,000 – 1,000,000 | A) 753 [150 – 1,560]  B) 1,570 [314 – 3,120]  C) 2,250 [509 – 4,280]  A) 6,220 [ 1,650 – 11,800]  B) 5,690 [1,200 – 11,800]  C) 1,870 [379 – 3,780]  A) 1,520 [554 – 2,900]  B) 3,230 [2,810 – 3,470]  C) 2,650 [1,560 – 3,400]  A) 5,390 [4,630 – 5,960]  B) 5,250 [4,580 – 5,920]  C) 5,260 [4,580 – 5,930]  A) 18,600 [11,300 – 31,600]  B) 12,700 [10,300 – 16,400]  C) 11,600 [10,200 – 13,400] |
|  |  |  |  |
| Mutation rate | Microsatellite –Stepwise Mutation Model (SMM) | 9x10^-06^-1x10^-05^ | A) 9.53 x10^-06^ [9.09 x10^-06^ – 9.98 x10^-06^]  B) 9.53 x10^-06^ [9.08 x10^-06^ – 9.98 x10^-06^]  C) 9.50 x10^-06^ [9.05 x10^-06^ – 9.95 x10^-06^] |
|  |  |  |  |
| Confidence | Type I error  (Simulated under scenario 1)  Type II error  (Simulated under scenario 2)  Type II error  (Simulated under scenario 3)  Type II error  (Simulated under scenario 4) | N/A  N/A  N/A  N/A | A) 0.294  B) 0.302  C) 0.320  A) 0.004  B) 0.006  C) 0.004  A) 0.300  B) 0.290  C) 0.314  A) 0.006  B) 0.000  C) 0.002 |

^1^ Time in generations (10 generations / year).

W: West

E: East
