# Supplementary material for: Population Genetic Analysis of Aedes aegypti Mosquitoes From Sudan Revealed Recent Independent Colonization Events by the Two Subspecies
Source: Front Genet. 2022 Feb 14;13:825652. doi: 10.3389/fgene.2022.825652 (PMC8889412; doi:10.3389/fgene.2022.825652)
Supplement: Supplementary file 9 [file Table6.DOCX]

**Table S6:** Summary of the genetic variation at 12 microsatellite loci by sampling location.

| Pop |  | AC1 | AC2 | AC4 | AC5 | CT2 | AG1 | AG2 | AG5 | A1 | A9 | B2 | B3 |
| --- | --- | --- | --- | --- | --- | --- | --- | --- | --- | --- | --- | --- | --- |
| Al Fashir | **N** | 50 | 50 | 50 | 50 | 50 | 50 | 50 | 50 | 50 | 50 | 49 | 47 |
|  | **Na** | 8 | 3 | 4 | 12 | 6 | 7 | 9 | 2 | 4 | 6 | 5 | 5 |
|  | **Ne** | 3.432 | 1.548 | 1.391 | 6.112 | 3.390 | 4.146 | 3.300 | 1.020 | 2.052 | 3.613 | 2.189 | 2.619 |
|  | **I** | 1.490 | 0.659 | 0.573 | 2.071 | 1.453 | 1.577 | 1.504 | 0.056 | 0.938 | 1.464 | 0.964 | 1.116 |
|  | **Ho** | 0.660 | 0.220 | 0.320 | 0.740 | 0.720 | 0.360 | 0.520 | 0.020 | 0.540 | 0.560 | 0.531 | 0.638 |
|  | **He** | 0.709 | 0.354 | 0.281 | 0.836 | 0.705 | 0.759 | 0.697 | 0.020 | 0.513 | 0.723 | 0.543 | 0.618 |
|  | **uHe** | 0.716 | 0.357 | 0.284 | 0.845 | 0.712 | 0.766 | 0.704 | 0.020 | 0.518 | 0.731 | 0.549 | 0.625 |
|  | **F** | 0.069 | 0.378 | -0.139 | 0.115 | -0.021 | **0.526** | 0.254 | -0.010 | -0.053 | 0.226 | 0.023 | -0.033 |
| Nyala | **N** | 48 | 48 | 48 | 48 | 48 | 48 | 48 | 48 | 48 | 47 | 48 | 47 |
|  | **Na** | 9 | 11 | 7 | 13 | 8 | 8 | 11 | 8 | 9 | 6 | 7 | 7 |
|  | **Ne** | 4.622 | 4.170 | 1.674 | 8.581 | 4.897 | 5.201 | 3.569 | 1.363 | 4.071 | 4.797 | 3.499 | 2.551 |
|  | **I** | 1.794 | 1.876 | 0.885 | 2.286 | 1.752 | 1.798 | 1.671 | 0.679 | 1.626 | 1.656 | 1.425 | 1.220 |
|  | **Ho** | 0.729 | 0.646 | 0.396 | 0.771 | 0.792 | 0.750 | 0.708 | 0.271 | 0.667 | 0.532 | 0.708 | 0.532 |
|  | **He** | 0.784 | 0.760 | 0.403 | 0.883 | 0.796 | 0.808 | 0.720 | 0.266 | 0.754 | 0.792 | 0.714 | 0.608 |
|  | **uHe** | 0.792 | 0.768 | 0.407 | 0.893 | 0.804 | 0.816 | 0.727 | 0.269 | 0.762 | 0.800 | 0.722 | 0.615 |
|  | **F** | 0.070 | 0.150 | 0.017 | 0.127 | 0.005 | 0.071 | 0.016 | -0.016 | 0.116 | 0.328 | 0.008 | 0.125 |
| Kassala | **N** | **52** | **52** | **52** | **52** | **52** | **52** | **52** | **52** | **52** | **52** | **52** | **52** |
|  | **Na** | 8 | 8 | 4 | 14 | 6 | 4 | 8 | 6 | 7 | 5 | 5 | 4 |
|  | **Ne** | 1.745 | 1.724 | 1.892 | 5.297 | 3.369 | 3.076 | 3.902 | 4.166 | 3.290 | 1.784 | 2.869 | 1.081 |
|  | **I** | 1.022 | 0.875 | 0.763 | 2.019 | 1.435 | 1.237 | 1.512 | 1.523 | 1.431 | 0.764 | 1.306 | 0.203 |
|  | **Ho** | 0.500 | 0.346 | 0.462 | 0.769 | 0.577 | 0.462 | 0.692 | 0.673 | 0.635 | 0.423 | 0.654 | 0.038 |
|  | **He** | 0.427 | 0.420 | 0.471 | 0.811 | 0.703 | 0.675 | 0.744 | 0.760 | 0.696 | 0.439 | 0.651 | 0.075 |
|  | **uHe** | 0.431 | 0.424 | 0.476 | 0.819 | 0.710 | 0.681 | 0.751 | 0.767 | 0.703 | 0.444 | 0.658 | 0.076 |
|  | **F** | -0.171 | 0.176 | 0.021 | 0.052 | 0.180 | 0.316 | 0.069 | 0.114 | 0.088 | 0.037 | -0.004 | **0.486** |
| Port Sudan | **N** | 51 | 51 | 51 | 51 | 51 | 51 | 51 | 51 | 51 | 51 | 51 | 51 |
|  | **Na** | 5 | 3 | 2 | 8 | 4 | 4 | 6 | 6 | 4 | 3 | 2 | 3 |
|  | **Ne** | 1.883 | 1.589 | 1.940 | 4.539 | 2.646 | 2.340 | 4.746 | 4.014 | 3.095 | 2.039 | 1.435 | 1.930 |
|  | **I** | 0.898 | 0.672 | 0.677 | 1.689 | 1.055 | 0.995 | 1.644 | 1.452 | 1.237 | 0.741 | 0.481 | 0.757 |
|  | **Ho** | 0.490 | 0.235 | 0.392 | 0.745 | 0.647 | 0.647 | 0.627 | 0.686 | 0.529 | 0.451 | 0.216 | 0.373 |
|  | **He** | 0.469 | 0.371 | 0.484 | 0.780 | 0.622 | 0.573 | 0.789 | 0.751 | 0.677 | 0.510 | 0.303 | 0.482 |
|  | **uHe** | 0.474 | 0.374 | 0.489 | 0.787 | 0.628 | 0.578 | 0.797 | 0.758 | 0.684 | 0.515 | 0.306 | 0.487 |
|  | **F** | -0.046 | 0.365 | 0.190 | 0.044 | -0.040 | -0.130 | 0.205 | 0.086 | 0.218 | 0.115 | 0.289 | 0.227 |

N, sample size for each population.

Na, number of different alleles

Ne, number of effective alleles.

I**,** Shannon’s information index.

Ho, observed heterozygosity.

He, expected heterozygosity.

F, Inbreeding coefficient representing the reduction of heterozygosity in a subpopulation due to non-random mating.

Bolded F value represent notable heterozygous deficits (F>0.50
